# Subacute Thyroiditis Masking as Fever of Unknown Origin: An Intriguing Case Report

**DOI:** 10.7759/cureus.43525

**Published:** 2023-08-15

**Authors:** Nishka Utpat, Vinod Nookala, Satyendra P Singh, Anil Sharma

**Affiliations:** 1 Internal Medicine, Rutgers Health/Community Medical Center, Newark, USA; 2 Internal Medicine, Community Medical Center, Toms River, USA

**Keywords:** hyperthyroidism, case report, abnormal thyroid function tests, subacute thyroiditis, fever of unknown origin

## Abstract

This study presents a unique case of subacute thyroiditis, which presents as prolonged high-grade fever without any other symptoms except for mild throat pain. A 46-year-old, otherwise healthy male presented with high-grade fever for more than two to three weeks and was found to have hyperthyroidism, with elevated free thyroxine levels (free T4), low thyroid-stimulating hormone (TSH) levels, elevated c-reactive protein (CRP) an inflammatory marker, and heterogeneous bilateral thyroid nodules on imaging studies. His workup was negative for infectious etiology of fever, thus favoring the diagnosis of subacute thyroiditis as the cause of fever of unknown origin (FUO). This case highlights the importance of considering subacute thyroiditis as a potential etiology in patients with FUO and the significance of a comprehensive workup to guide appropriate management.

## Introduction

Hyperthyroidism is a condition where the thyroid gland produces excessive amounts of thyroid hormones, resulting in an overactive metabolism. Common symptoms include weight loss, increased appetite, rapid heartbeat, irritability, and difficulty sleeping [1]. The most common cause of hyperthyroidism is an autoimmune disorder called Graves' disease, but it can also be caused by thyroid nodules or inflammation, presenting as subacute thyroiditis [2]. Subacute thyroiditis is essentially a clinical diagnosis but blood tests to measure levels of thyroid hormones are helpful in times of diagnostic dilemma and for confirmation. The treatment aims to manage the symptoms and stabilize thyroid function. Treatment options include medication to suppress thyroid hormone production, radioactive iodine therapy to destroy thyroid cells, or surgery to remove the thyroid gland.

Follow-up lab tests are needed to monitor the thyroid hormone levels as hyperthyroidism can lead to hypothyroidism after going through a phase of normalization of thyroid hormone levels [3]. Consultation with endocrinology and follow-up is prudent to manage this condition as there is a risk of progression to hypothyroidism, thus mandating monitoring of the thyroid hormone levels on a periodic basis.

## Case presentation

Here, we present a case of a 46-year-old male who presented with approximately two to three weeks of high-grade fever, fatigue, dry cough, and mild throat pain without a sore throat or difficulty swallowing. Although his cough improved symptomatically with over-the-counter cough syrup, he had daily fevers despite antipyretics. Additionally, he had received a course of oral levofloxacin through his primary care physician, for a presumptive upper respiratory tract infection. Otherwise, he was not on any medications upon presentation. He continued to have a fever and presented to our hospital due to failure of oral antibiotics as an outpatient and was hospitalized for a fever of unknown origin (FUO). He denied any gastrointestinal, genitourinary tract, musculoskeletal, or integumentary symptoms. Vital signs were relevant for a temperature of 102.8°F and a pulse rate of 121 beats per minute. Respiratory rate and blood pressure were within normal limits indicating hemodynamic stability. Except for a mild tenderness on palpation of the anterior part of the neck, physical examination, and routine labs were unremarkable (Table 1).

**Table 1:** Initial complete blood count. WBC: white blood cell count; RBC: red blood cell count; Hgb: hemoglobin; Hct: hematocrit

Labs	Complete blood count		
Date	April 8, 2022	Units	Reference range
WBC	11.4	×10^3^/µL	4.80-10.80
RBC	4.61	×10^6^/µL	4.20-5.40
Hgb	11.8	g/dL	12.0-16.0
Hct	35.9	%	37.0-47.0
Platelet	320	×10^3^/µL	130-400

Given the prolonged fever with no apparent source of infection, a consultation by an infectious diseases specialist was sought who initiated extensive workup for FUO. Initial labs were suggestive of mild leukocytosis, elevated liver function tests, and inflammatory markers (Table 2). Microbiology studies were done and reported as negative (Table 3). Autoimmune disorders, malignancies, and systemic inflammatory diseases were explored with unremarkable results (Table 4). Appropriate tests were also done for viral pathogens, tick-borne and travel-related illnesses, tuberculosis, endocarditis, and abscesses with negative results (Table 5).

**Table 2:** Initial and subsequent liver function tests. ALT: alanine aminotransferase; AST: aspartate aminotransferase; Alk Phos: alkaline phosphatase

Labs	Liver function tests			
Date	April 8, 2022	April 10, 2022	Units	Reference range
ALT	122	114	U/L	7-40
AST	82	58	U/L	15-40
Alk Phos	120	113	U/L	45-117

**Table 3:** Initial microbiology tests. PCR: polymerase chain reaction; RSV: respiratory syncytial virus

Labs (dated: April 8, 2022)	Results
Urine culture	Negative
Blood cultures ×2	Negative
Flu/RSV PCR	Negative
Respiratory pathogen panel by PCR	Negative
Hepatitis panel	Non-reactive

**Table 4:** Various tests done for autoimmune diseases. ANA: antinuclear antibody; RF Qnt: rheumatoid factor quantitative level

Labs	Dates			Reference range
	April 8, 2022	April 12, 2022	April 13, 2022	
ESR	62	N/A	129	0-22 mm/h
CRP	89.30	N/A	54.10	8-10 mg/L
ANA	N/A	Negative	N/A	Negative
RF Qnt	N/A	>14	N/A	<15 IU/mL

**Table 5:** Travel and tick-borne illness workup.

Labs	Dates		Reference range
	April 8, 2022	April 11, 2022	
Malaria smear	N/A	None seen	No parasites seen=negative
Lyme disease screen	<0.90 negative	N/A	<0.9=negative; 0.91-1.09=equivocal; >1.1=positive
Anaplasma IgG/IgM	Negative	N/A	IgG<1.64: negative; IgM<1.20: negative
Ehrlichia IgG/IgM	Negative	N/A	IgG<1.64: negative; IgM<1.20: negative
Babesia IgG/IgM	Negative	N/A	IgG<1.64: negative; IgM<1.20: negative
Rocky Mountain spotted fever IgG/IgM	Negative	N/A	IgG<1.64: negative; IgM<1.20: negative

**Figure 1:**
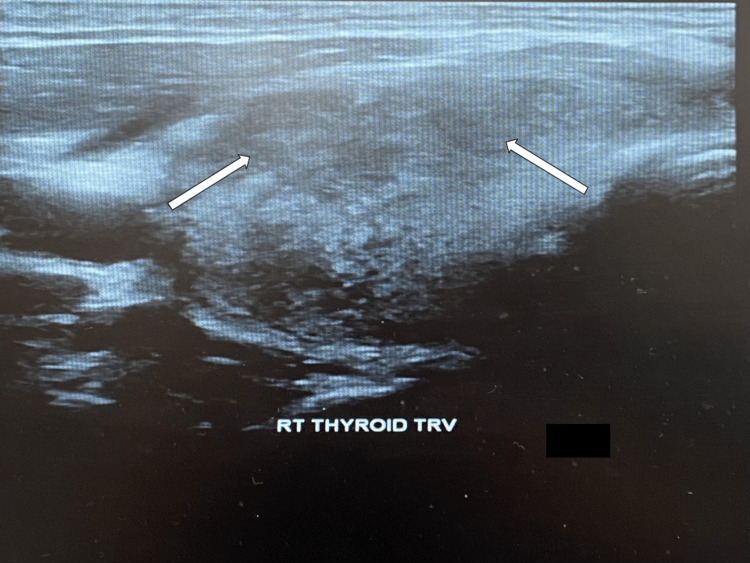
Ultrasound of enlarged heterogeneous thyroid with bilateral multiple nodules.

**Figure 2:**
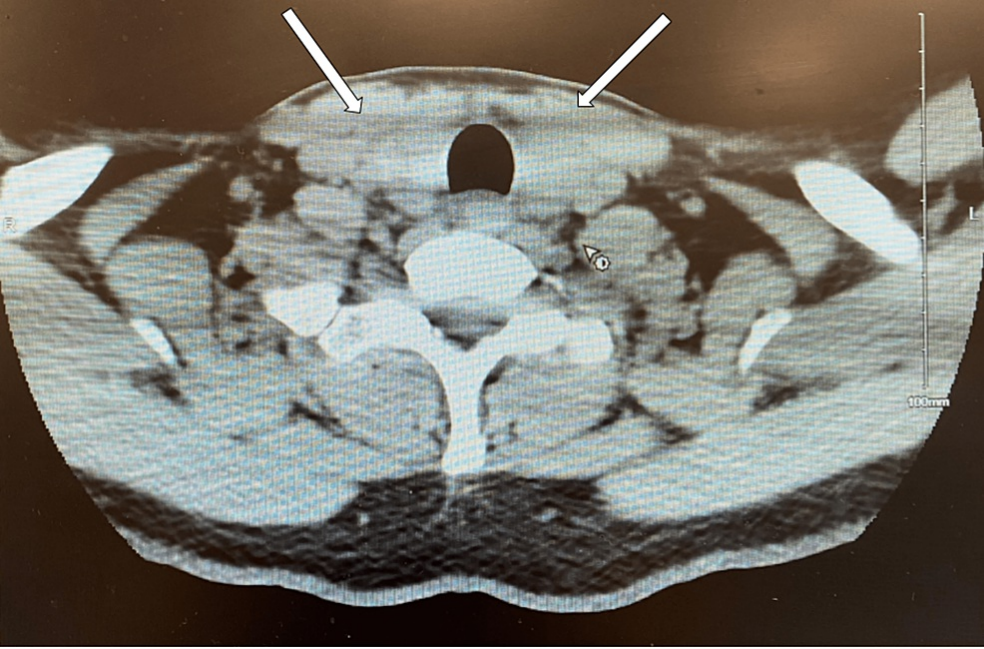
CT neck showing heterogeneous and prominent thyroid nodules.

**Figure 3:**
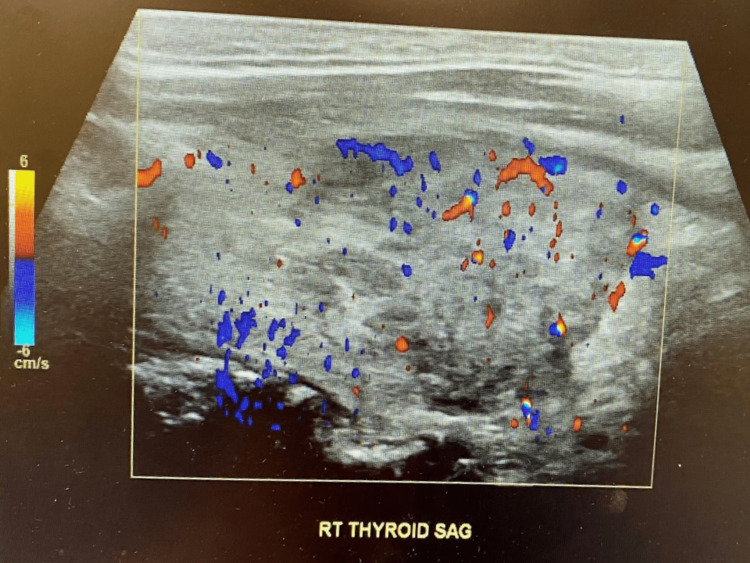
Ultrasound of enlarged heterogeneous thyroid with colored stills.

As a part of workup for tachycardia, thyroid function tests were done and found to be abnormal with low levels of thyroid stimulating hormone and high levels of free thyroxine hormone (Table 6). Previous lab work done by his primary physician was requested and compared to current labs, revealing normal thyroid-stimulating hormone (TSH), two months prior. Thyroid ultrasound showed enlarged heterogeneous thyroid with bilateral multiple nodules (Table 7 and Figure 1). CT neck showed heterogeneous and prominent thyroid (Table 7 and Figures 2, 3). A biopsy was deferred at this time, as the labs and imaging studies were highly suggestive of an inflammatory condition. A diagnosis of subacute thyroiditis was established.

**Table 6:** Initial and subsequent thyroid function tests. TSH: thyroid-stimulating hormone

Labs	Dates				Reference range
	April 12, 2022	April 13, 2022	April 14, 2022	3 months later	
TSH	N/A	<0.01	0.02	2.8	0.5-5.0 mIU/L
T4 free	2.93	3.05	N/A	8.3	5.0-12.0 μg/dL
T3 free	N/A	8.3	8.0	3.1	2.3-4.2 pg/mL

**Table 7:** Pertinent radiologic studies. RAIU: radioiodine thyroid uptake

Date	Type of study	Impression	Reference range
April 13, 2022	CT soft tissues neck without contrast	Heterogeneous prominent thyroid gland, suggestive of subacute thyroiditis	N/A
April 14, 2022	US thyroid	Large solid heterogeneous bilateral thyroid nodules	N/A
April 19, 2022	RAIU thyroid scan	Markedly diminished, thyroid uptake values at 4 h and 24 h (range: 4 h uptake: 0.8%; 24 h uptake: 0.2%)	3-16% at 4 h; 8-25% at 24 h

**Figure 4:**
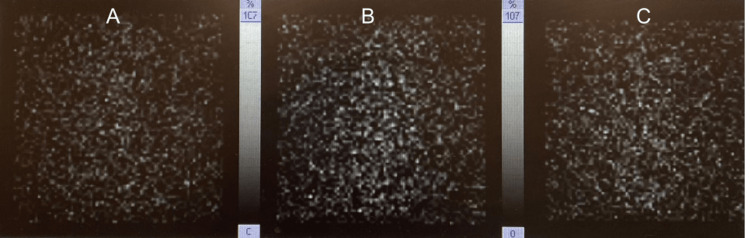
Radioactive Iodine uptake scan used to diagnose thyroid problems. The images show (A) radioactive iodine uptake at 0 h, (B) radioactive iodine uptake at 4 h, and (C) radioactive iodine uptake at 24 h.

He was discharged on aspirin and propranolol for symptom management. An appointment with endocrinology as an outpatient was made prior to his hospital discharge. A radioactive thyroid uptake imaging was done two weeks post-discharge and compared with the CT neck done during inpatient hospitalization. It showed markedly diminished thyroid uptake values at 4 h and 24 h, suggestive of late-stage subacute thyroiditis (Figure 4).

He was started on oral prednisone with slow taper by the endocrinologist. The patient's fever and tachycardia resolved soon after initiating treatment, and he reported significant improvement in his overall symptoms during the follow-up visits with normalization of thyroid hormone levels at a three-month follow-up.

## Discussion

The thyroid gland is responsible for many metabolic functions in the body like heart rate, lipid metabolism, and skeletal growth [4]. Subacute thyroiditis is an inflammatory condition that can manifest with a variety of clinical presentations. In most patients, clinical manifestations, like neck pain, significant thyroid tenderness, and presence of goiter are sufficient for diagnosis. While it is commonly associated with symptoms of hyperthyroidism or hypothyroidism, fundamentally it is a clinical diagnosis. When clinical presentations are subtle or unusual, it is challenging to diagnose this condition clinically. It can also present as an isolated prolonged fever, mimicking FUO. Hyperthyroidism is transient, lasting from two to eight weeks, and may be followed by a period of transient, usually asymptomatic, overt, or subclinical hypothyroidism. This study emphasizes the importance of considering subacute thyroiditis in the differential diagnosis of FUO, particularly when there are associated symptoms, such as tachycardia, fatigue, and a tender thyroid gland. Prompt recognition and appropriate management of subacute thyroiditis can lead to the resolution of symptoms and prevent unnecessary diagnostic tests and treatments.

Most likely the patient’s upper respiratory tract illness had affected the thyroid gland and triggered subsequent subacute thyroiditis [5]. Only a subtle neck pain and minimal to no tenderness of the anterior part of the neck without palpable enlargement made this case challenging [6]. With limited consulting services and a lack of in-house specialty labs in a small community hospital, the rapid workup and diagnosis of such a condition depends on keen acuity of internists and infectious diseases specialists if involved. This case highlights the recognizable and collaborative work done by physicians at small community hospitals.

## Conclusions

Subacute thyroiditis should be considered in the differential diagnosis of FUO, especially when associated with a palpable, even minimally tender thyroid gland and unexplained tachycardia with constitutional symptoms. A comprehensive evaluation, collaboration of involved physicians, timely and appropriate lab work, and comparison with previous labs if available, especially thyroid function tests and imaging studies, are crucial to establish the diagnosis. Early recognition and management of thyroiditis can lead to favorable outcomes and avoid unnecessary investigations. Further studies are warranted to enhance our understanding of the relationship between subacute thyroiditis, upper respiratory viral illnesses, and FUO, which in turn will help to improve diagnostic approaches and management strategies.
